# Patterns of brain metastasis immediately before prophylactic cranial irradiation (PCI): implications for PCI optimization in limited-stage small cell lung cancer

**DOI:** 10.1186/s13014-019-1371-4

**Published:** 2019-09-18

**Authors:** Xiao Chu, Shuyan Li, Bingqing Xia, Li Chu, Xi Yang, Jianjiao Ni, Liqing Zou, Yida Li, Congying Xie, Jie Lin, Zhengfei Zhu

**Affiliations:** 1 Department of Radiation Oncology, Fudan University Shanghai Cancer Center, Fudan University, 270 DongAn Road, XuHui District, Shanghai, 200032 China; 2 Department of Oncology, Shanghai Medical College, Fudan University, Shanghai, 200032 China; 3 Department of Radiology, Fudan University Shanghai Cancer Center, Fudan University, Shanghai, 200032 China; 4 Radiotherapy and Chemotherapy Department, the 1st Affiliated Hospital of Wenzhou Medical University, Wenzhou, 325000 Zhejiang China; 5 Department of Medical Oncology, the Second Affiliated Hospital of Kunming Medical University, 374 Dianmian Avenue, Wuhua District, Kunming, 650101 Yunnan China

**Keywords:** Limited-stage small cell lung cancer, Prophylactic cranial irradiation, Chemoradiotherapy duration, Brain metastasis

## Abstract

**Background:**

Prophylactic cranial irradiation (PCI) is indicated for limited-stage small cell lung cancer (LS-SCLC) with good response to chemoradiotherapy (CRT). However, brain metastasis (BM) developed in LS-SCLC before PCI is not rare. In this study, we comprehensively investigated the features of pre-PCI BMs, aiming to explore the potential of PCI optimization for LS-SCLC.

**Methods:**

One-hundred-ten LS-SCLC patients achieving clinical complete remission after definitive CRT with contrast-enhanced cranial magnetic resonance imaging (MRI) at baseline and immediately before PCI were included. The time trend and risk factors for pre-PCI BM were evaluated. Several radiological features, including numbers, sizes, and locations of pre-PCI BMs, were investigated to explore the technical feasibility of stereotactic radiotherapy and hippocampal-avoidance (HA) PCI.

**Results:**

Twenty-four (21.8%) of the LS-SCLC patients harbored pre-PCI BM, all except one were asymptomatic. CRT duration (CRT-D) was the only independent risk factor for pre-PCI BM. The pre-PCI BM rate gradually increased in line with a growing time interval between treatment initiation and pre-PCI MRI. Pre-PCI BM and prolonged CRT-D were both correlated with worse overall survival. Of 129 pre-PCI intracranial lesions, 2 (1.5%) were in the HA region. Eight of the 24 (33.3%) pre-PCI BM patients were ineligible for stereotactic radiotherapy.

**Conclusion:**

Our findings suggest that PCI is still of importance in LS-SCLC, and MRI evaluation before PCI is indispensable. Investigations are warranted to explore the possibility of moving PCI up to before CRT completion in LS-SCLC patients with prolonged CRT-D. HA-PCI could be considered to reduce neurotoxicity.

**Electronic supplementary material:**

The online version of this article (10.1186/s13014-019-1371-4) contains supplementary material, which is available to authorized users.

## Introduction

Small cell lung cancer (SCLC) features a rapid doubling time and an aggressive behavior. Brain is a favored site of metastasis in SCLC, and the 2-year brain metastasis (BM) rate could reach more than 50% – even in SCLC patients who achieved complete remission (CR) after chemotherapy [1].

Prophylactic cranial irradiation (PCI) has been proved to effectively reduce BM and improve survival in limited-stage SCLC (LS-SCLC) patients with a good response to definitive chemoradiotherapy (CRT) [1–4]. Theoretically, there could be some patients experiencing BM before PCI, due to poor penetration of drugs through the blood-brain barrier. However, most previous studies exploring the value of PCI in SCLC did not enforce brain imaging immediately before PCI [1, 2]. A small-cohort study, employing cranial magnetic resonance imaging (MRI) immediately before PCI in LS-SCLC patients who had achieved CR after CRT, found that 32.5% of the patients developed BM before PCI [5].

Here, we report the prevalence of and risk factors for BM immediately before PCI (pre-PCI BM), based on contrast-enhanced cranial MRIs in a larger cohort from our center. We also investigated the time trend for pre-PCI BM and several radiological features of the intracranial lesions, aiming to explore the potential of PCI optimization for LS-SCLC patients in the MRI era.

## Patients and methods

### Patients

Patients with histologically or cytologically confirmed SCLC at Fudan University Shanghai Cancer Center between 2011 and 2017 were reviewed. The initial staging was based on the Veterans Administration Lung Study Group two-stage classification and a modified staging system proposed by the International Association for the Study of Lung Cancer [6, 7]. 283 LS-SCLC patients who were treated with definitive CRT and received baseline contrast-enhanced cranial MRI were identified. Tumor response to CRT was assessed using Response Evaluation Criteria in Solid Tumors version 1.1. Among the 283 patients, 110 achieved clinical CR after CRT and had a second contrast-enhanced cranial MRI immediately before the scheduled PCI; these patients formed the cohort of interest (Additional file 1: Figure S1). The clinical characteristics of the cohort are listed in Table 1. The median follow-up time for this cohort is 22.7 months (range 6.4–92.0 months).

**Table 1:** Patients’ clinical characteristics

Characteristic	Patient number (*n* = 110)	Proportion (%)
Gender		
Male	95	86.4%
Female	15	13.6%
Smoking status		
ever-smokers	86	78.2%
never-smokers	24	21.8%
T stage		
1	21	19.1%
2	27	24.5%
3	34	30.9%
4	28	25.5%
N stage		
0	2	1.8%
1	13	11.8%
2	73	66.4%
3	22	20.0%
Pre-PCI BM		
Positive	24	21.8%
Negative	86	78.2%
	Range	Median
Age at diagnosis (years)	38–79	60
CRT-D (months)	2.2–8.1	4.1

*Abbreviation*: *PCI* Prophylactic cranial irradiation, *BM* Brain metastasis, evaluated bycranial magnetic resonance imaging, *CRT-D* Chemoradiotherapy duration. T and Nstaging was based on the 8th TNM staging system for lung cancer

### Definitive CRT

Most of the patients received 4 to 6 cycles of chemotherapy using etoposide plus cisplatin or carboplatin, only 2 patients received irinotecan plus cisplatin. Definitive thoracic radiotherapy (TRT) was delivered concurrently (at least 2 cycles of chemotherapy delivered during TRT, n = 93) or sequentially (n = 17) with chemotherapy. The TRT schedules included conventional-fractionated (2 Gy daily, DT ≥ 56 Gy, n = 94), hyper-fractionated (1.5 Gy twice daily, DT = 45 Gy, n = 4), and hypo-fractionated (2.5 Gy daily, DT = 55 Gy, n = 12) [8] plans. TRT was delivered with intensity-modulated radiotherapy or three-dimensional conformal radiotherapy techniques.

### Contrast-enhanced cranial MRI

All of the cranial MRIs were performed at our center, following a routine schedule as described here. The images obtained before administration of the contrast agent (gadolinium) were axial T1- and T2-weighted images (6 mm sections, 1 mm gap), and sagittal T1-weighted images (4 mm sections, contiguous). The images obtained after administration of the contrast agent were axial and coronal thin sections (4 mm, contiguous) that were T1-weighted (fast spoiled gradient recalled echo acquisition, TR 120–215, TE 2.2–2.8). All MRI evaluations were carried out by the same team of clinical imaging technicians and neuroradiologists and were performed on the same 1.5 Tesla MRI machine (GE Healthcare, Waukesha, WI). In the present cohort the median time between CRT initiation and pre-PCI MRI was 5.2 months (range 2.8–9.2 months).

### Brain irradiation

PCI (25 Gy in 10 fractions daily) was performed in all 86 patients who did not experience pre-PCI BM. The median time interval between pre-PCI MRI and PCI is 12 days (range 2–35 days). The patients (n = 24) with pre-PCI BM received whole-brain radiotherapy (WBRT) (30 Gy in 10 fractions daily).

### Radiological features of the pre-PCI BMs

To evaluate the feasibility of hippocampus-avoidance (HA) PCI for SCLC, the hippocampus was delineated on MRI images in the patients with pre-PCI BM, according to the Radiation Therapy Oncology Group 0933 protocol [9]. All BM lesions were documented according to their central locations from the hippocampus. Hippocampal metastasis was defined as when the lesion’s center was located within 5 mm of the hippocampus. The number of lesions and diameter thereof were documented. We also evaluated the technical feasibility of stereotactic radiotherapy (SRT) for the pre-PCI BM patients, based on the eligibility criteria of the Japanese Leksell Gamma Knife (JLGK) Society 0901 study [10]. These criteria include: ten or fewer brain metastases; largest tumor < 10 mL in volume and <  3.0 cm in longest diameter; cumulative volume of all tumors < 15.0 mL; and no evidence of leptomeningeal dissemination.

### Statistics

Continuous variables were summarized by descriptive statistics, such as standard deviations, medians, and ranges. Categorical variables were tabulated by frequency and percentage. Binary logistic regression was employed to evaluate risk factors for pre-PCI BM. The prognostic significance of multiple clinical variables was evaluated via Cox proportional hazards regression. Variables with a *P* value of less than 0.1 in univariate analyses were included in multivariate analyses, while tumor stage and lymph node stage were included irrespective of their univariate *P* value.

The prognostic significance of CRT duration (CRT-D) and pre-PCI BM were calculated in two independent multivariate analyses. Kaplan-Meier plots were used to visualize the event-time distributions in survival analysis, and differences between groups were compared via log-rank test. All statistical tests were two-sided, and *P* values of less than 0.05 were considered statistically significant. Statistical Package for Social Sciences (SPSS version 20.0, IBM, NY, USA) software was used for all statistical analyses. Kaplan-Meier plots were generated via GraphPad Prism 6 (GraphPad Software, CA, USA).

## Results

### Prevalence, risk factors and time trend of pre-PCI BM in LS-SCLC patients

Contrast-enhanced cranial MRI revealed that 24 (21.8, 95% confidence interval [CI] 14.0–29.7%, Table 1) out of the 110 LS-SCLC patients in clinical CR harbored pre-PCI BM. Only one of these patients was neurologically symptomatic at the time of pre-PCI MRI evaluation. The time interval from CRT completion to pre-PCI MRI was not significantly different between patients with and without pre-PCI BM.

We subsequently performed risk factor analysis on pre-PCI BM, including age, gender, smoking status, tumor stage, lymph node stage and CRT-D, via binary logistic regression. CRT-D was defined as the time interval between CRT initiation and completion. The median of CRT-D was 4.1 months (range 2.2–8.1 months). The risk factor analysis showed that only CRT-D is an independent risk factor for pre-PCI BM: longer CRT-D was correlated with higher pre-PCI BM prevalence (risk ratio 1.406, 95% CI 1.007–1.964, *P* = 0.045, Table 2).

**Table 2:** Risk factor analysis for pre-PCI brain metastasis

Covariates	Univariate		Multivariate	
	RR (95% CI)	*P*	RR (95% CI)	*P*
Age at diagnosis	0.976 (0.924–1.032)	0.400		
Gender	0.510 (0.107–2.437)	0.399		
Smoking	3.781 (0.822–17.400)	0.088	4.376 (0.895–21.394)	0.068
T stage (1–2 vs 3–4)	1.383 (0.546–3.502)	0.494	1.099 (0.411–2.941)	0.851
N stage (0–2 vs 3)	1.458 (0.499–4.259)	0.490	1.389 (0.456–4.235)	0.564
CRT-D (months)	1.340 (0.981–1.831)	0.066	1.406 (1.007–1.964)	0.045*

*Abbreviation*: *PCI* Prophylactic cranial irradiation, *RR* Risk ratio, *CI* Confidence interval, *CRT-D* Chemoradiotherapy duration. Age and CRT-D as continuous variables. Binary logistic regression was employed for analysis. (*) statistically significant

**Fig. 1:**
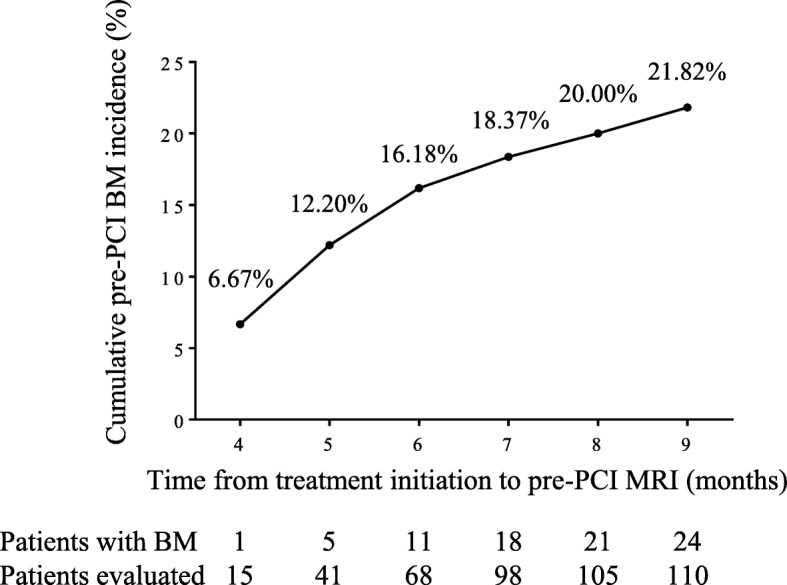
Cumulative incidence of pre-PCI BM. The cumulative incidence of pre-PCI BM is shown according to the time interval between treatment initiation and pre-PCI MRI. The bottom number shows the number of patients evaluated and patients with pre-PCI BM detected at different time points

We also analyzed the cumulative incidence of pre-PCI BM according to the time interval between treatment initiation and pre-PCI MRI, which showed pre-PCI BM rate gradually increased as this time interval lengthened (Fig. 1). The steepest ascent was embodied at the time from fourth to fifth months: at the fourth month, the pre-PCI BM rate was 6.67% (1 out of 15), while at the fifth month it was 12.20% (5 out of 41) (Fig. 1).

### Impact of pre-PCI BM and CRT-D on survival

**Fig. 2:**
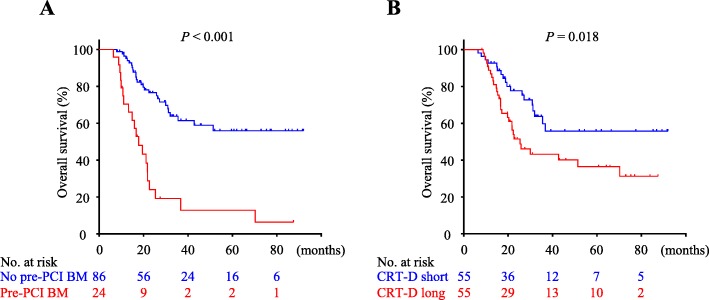
Impact of pre-PCI BM and CRT duration on survival. Kaplan-Meier curves of overall survival (OS) based on (**a**) pre-PCI brain metastasis (BM) and (**b**) CRT duration (CRT-D, 4.1 months or longer versus 4.0 months or shorter). The bottom numbers indicate patients at risk at different time points

Cox proportional hazard regression was performed to evaluate the risk factors for overall survival (OS). Both the pre-PCI BM and CRT-D were shown to be associated with OS on the univariate analysis. Since a correlation existed between pre-PCI BM and CRT-D, we included them in two separate multivariate analyses. We found pre-PCI BM and CRT-D to be independent prognostic factors for OS in each analysis (Table 3). The event-time distributions in survival analysis were visualized in Kaplan-Meier plots (Fig. 2).

**Table 3:** Multivariate analyses for overall survival

Variable	Univariate		Multivariate	
	HR (95% CI)	*P*	HR (95% CI)	*P*
Age at diagnosis	1.022 (0.986–1.059)	0.235		
Gender	1.725 (0.728–4.086)	0.215		
Smoking status	1.205 (0.614–2.366)	0.588		
T stage (1–2 vs 3–4)	2.247 (1.215–4.157)	0.010*	2.610 (1.364–4.993)	0.004*
N stage (0–2 vs 3)	1.513 (0.771–2.968)	0.229	2.160 (1.056–4.417)	0.035*
CRT-D (months)	1.219 (1.024–1.451)	0.026*	1.227 (1.026–1.466)	0.025*
Variable	HR (95% CI)	*P*	HR (95% CI)	*P*
Age at diagnosis	1.022 (0.986–1.059)	0.235		
Gender	1.725 (0.728–4.086)	0.215		
Smoking status	1.205 (0.614–2.366)	0.588		
T stage (1–2 vs 3–4)	2.247 (1.215–4.157)	0.010*	2.637 (1.348–5.158)	0.005*
N stage (0–2 vs 3)	1.513 (0.771–2.968)	0.229	2.016 (0.967–4.202)	0.061
pre-PCI BM	4.336 (2.421–7.765)	< 0.001*	4.143 (2.318–7.406)	< 0.001*

*Abbreviation*: *HR* Hazard ratio, *CI* Confidence interval, *PCI* Prophylactic cranial irradiation, *BM* Brain metastasis, *CRT-D* Chemoradiotherapy duration, *pre-PCI BM* Brain metastasis immediately before PCI. Age and CRT-D as continuous variables. Cox regression was employed for multivariate analysis. (*) statistically significant

### Radiological features of the pre-PCI brain lesions

The radiological features of the pre-PCI BMs in 24 patients are shown in Table 4. Of these patients, 14 (58.3%) had multiple intracranial lesions; 12 (50%) had 4 or more intracranial lesions. There was a total of 129 pre-PCI intracranial lesions, with a median diameter of 8 mm (range 3–62 mm). Of these lesions, only 2 were located in the HA region (1.5, 95% CI 0.4–5.2%); these 2 patients with HA region metastasis had 5 and 28 pre-PCI intracranial lesions, respectively (Table 4).

**Table 4:** Radiological features of pre-PCI brain metastases

Eligibility criteria for stereotactic radiotherapy					
Patient #	Total no. of lesions (1 to 10)	Longest diameter (< 3 cm)	Largest lesion volume (< 10 mL)	Total tumor volume (< 15 mL)	No. of lesions in the HA region
#1	1	1.5	1.90	1.90	0
#2	1	0.5	0.10	0.10	0
#3	1	1.0	0.51	0.51	0
#4	1	0.8	0.33	0.33	0
#5	1	1.7	2.58	2.58	0
#6	1	1.2	0.97	0.97	0
#7	1	1.3	1.26	1.26	0
#8	1	0.7	0.20	0.20	0
#9	1	2.0	4.79	4.79	0
#10	1	2.4	7.54	7.54	0
**#11**	3	**3.0**	**13.44**	**21.94**	0
**#12**	3	**6.2**	**109.88**	**135.37**	0
#13	4	1.0	0.57	0.90	0
**#14**	4	**3.0**	**14.27**	**24.82**	0
#15	4	1.7	2.86	3.20	0
#16	4	1.6	2.24	3.75	0
**#17**	5	**3.1**	**16.20**	**33.35**	1
#18	6	1.3	1.19	5.08	0
#19	6	0.6	0.15	0.46	0
#20	7	2.3	6.38	11.90	0
**#21**	9	2.9	**12.57**	**43.19**	0
**#22**	**12**	1.8	3.16	**15.69**	0
**#23**	**24**	1.0	0.55	4.54	0
**#24**	**28**	0.9	0.39	5.85	1

The eligibility criteria was adapted from JLGK0901 study (reference #10). Bold face indicates patients ineligible for stereotactic radiotherapy and the specific criteria they failed to meet

*Abbreviation*: *PCI* Prophylactic cranial irradiation, *HA region* Hippocampal avoidance region

For SRT feasibility evaluation, the JLGK0901 inclusion criteria were employed [10], which identified 33.3% (8 out of 24) of the pre-PCI BM patients as not eligible for SRT. The specific reasons for their ineligibility are indicated in Table 4.

## Discussion

Our study corroborates the findings of a previous study in a relatively small cohort [5], which showed that there was a considerable risk of pre-PCI BM in LS-SCLC patients who achieved clinical CR after definitive CRT. Moreover, in both studies patients with pre-PCI BM were rarely neurologically symptomatic. These findings suggest the importance of MRI evaluation immediately before PCI, even in asymptomatic patients. In addition, we investigated the time trend and risk factors for pre-PCI BM and several image features of the intracranial lesions, which were not reported in the previous study [5]. Our findings indicate that some aspects could be optimized in PCI for LS-SCLC.

Treatment duration was often employed to represent the CRT time-intensity and has been revealed as an independent risk factor for OS in SCLC [11]. In the present study, longer CRT-D was found to be a risk factor for poor survival. The inferior survival of patients with longer CRT-D may have been the result of worse performance status, larger tumor burden and poorer compliance. However, it is also plausible that the worse survival in patients with longer CRT-D may be partly derived from a higher pre-PCI BM rate: in this study longer CRT-D was an independent risk factor for pre-PCI BM.

Many studies showed that BM incidence plateaued around 2 years after PCI for SCLC and remained significantly lower than control groups [1–3, 12]. This phenomenon indicates that the emergence of detectable BMs can be prevented, instead of simply delayed, by PCI. In other words, PCI is a curative rather than palliative therapy for subclinical BMs in SCLC. However, when these BMs become clinically detectable, PCI may only achieve palliative results. As shown in this study, although all of the patients with MRI detected pre-PCI BM received WBRT, the outcomes were significantly worse than in those without pre-PCI BMs.

In the current study, the cumulative incidence of MRI-detected pre-PCI BMs gradually increased along with the time interval between the treatment initiation and pre-PCI MRI, even though clinical CR was achieved for the extracranial disease. We speculate that with the longer treatment courses, there is more time for occult brain lesions to develop and become MRI-detectable, possibly due to the poor penetration of chemotherapy agents through the blood-brain barrier.

Considering all the evidence, we believe that the timing of PCI is crucial for optimal BM prevention. At present, 4–6 cycles of chemotherapy at 21-day intervals concurrently with TRT was recommended for LS-SCLC [13, 14], which would take 2–3.5 months if administered on schedule. In this study, the cumulative incidence of pre-PCI BM was 6.67% at the fourth month after initial treatment, which increased to 12.20% only 1 month later; it gradually increased further to 21.82% at the ninth month from treatment initiation. Thus, in patients who completed CRT on schedule, the pre-PCI BM risk is relatively low, and they can receive PCI after CRT completion. However, for patients whose CRT courses are prolonged to more than 4 months, postponing PCI further may expose them to higher risk of pre-PCI BM. Investigations of moving PCI up and before CRT completion in these patients are warranted.

Nonetheless, the timing of PCI should be weighed against neurotoxicity, because neurocognitive deficits have been reported to be more severe when PCI is delivered concurrently with chemotherapy [15, 16]. The HA technique was found to better preserve neurocognitive functions in WBRT [17, 18], and thus has garnered increasing interest recently. However, the intracranial seeding pattern immediately before PCI was previously unreported. Therefore, we investigated the risk of pre-PCI BMs in the HA region, to explore the safety of HA-PCI for LS-SCLC patients. In contrast to a previous study [19], our cohort is more likely to represent the pattern of de novo LS-SCLC BM distribution owing to the pre-PCI MRI, and that all patients included had limited diseases outside the brain. Consistent with the previous study [19], our results also showed that the risk of BM in the HA region is low.

SRT is a curative approach with lower toxicity for BMs from solid tumors, which is extensively employed in clinical practice. The idea of reserving PCI in LS-SCLC, and using salvage SRT when BMs emerge, had been raised [20]. In the present study, we investigated the image features of the intracranial lesions to explore the technical feasibility of applying SRT for the BMs detected before PCI. However, there are no standard criteria for BM SRT at present. BM numbers of 1–3 or 4 were traditionally thought to be most suitable, but there is increasing evidence to support that SRT could benefit patients with more than 4 BMs. The present highest-level evidence comes from the multi-institutional prospective observational study conducted by the JLGK Society (JLGK0901) [10], which demonstrated that the therapeutic effect of SRT in patients with 5–10 BMs is comparable to that in those with 2–4 BMs. We employed the JLGK0901 eligibility criteria for evaluation of SRT feasibility in this study and found that 33.3% of the patients did not meet the criteria. This indicates that it is not appropriate to abandon PCI in these patients.

## Conclusions

In summary, our findings suggest that PCI is still of importance for LS-SCLC, and patients should undergo MRI evaluation immediately before PCI. Studies exploring the possibility of moving PCI up and before CRT completion in LS-SCLC patients with prolonged CRT-D, and the feasibility of HA-PCI to reduce neurotoxicity, are warranted.

## Additional file

Additional file 1: **Figure S1.** Patient selection workflow. Selection of LS-SCLC patients treated at Fudan University Shanghai Cancer Center (FUSCC) according to the response to definitive chemoradiotherapy (CRT) and the findings of the contrast-enhanced cranial MRI performed immediately before PCI. (PDF 12 kb)

## Data Availability

The datasets used and/or analyzed during the current study are available from the corresponding author on reasonable request.

## Abbreviations

BM: Brain metastasis
CI: Confidence interval
CR: Complete remission
CRT: Chemoradiotherapy
CRT-D: Chemoradiotherapy duration
HA: Hippocampal avoidance
LS-SCLC: Limited-stage small cell lung cancer
MRI: Magnetic resonance imaging
PCI: Prophylactic cranial irradiation
SRT: Stereotactic radiotherapy
TRT: Thoracic radiotherapy
WBRT: Whole-brain radiotherapy
