# Supplementary material for: Patterns of brain metastasis immediately before prophylactic cranial irradiation (PCI): implications for PCI optimization in limited-stage small cell lung cancer
Source: Radiat Oncol. 2019 Sep 18;14:171. doi: 10.1186/s13014-019-1371-4 (PMC6749639; doi:10.1186/s13014-019-1371-4)
Supplement: Supplementary file 1 — Figure S1. Patient selection workflow. Selection of LS-SCLC patients treated at Fudan University Shanghai Cancer Center (FUSCC) according to the response to definitive chemoradiotherapy (CRT) and the findings of the contrast-enhanced cranial MRI performed immediately before PCI. (PDF 12 kb) [file 13014_2019_1371_MOESM1_ESM.pdf]

**Supplemental Figure 1.**

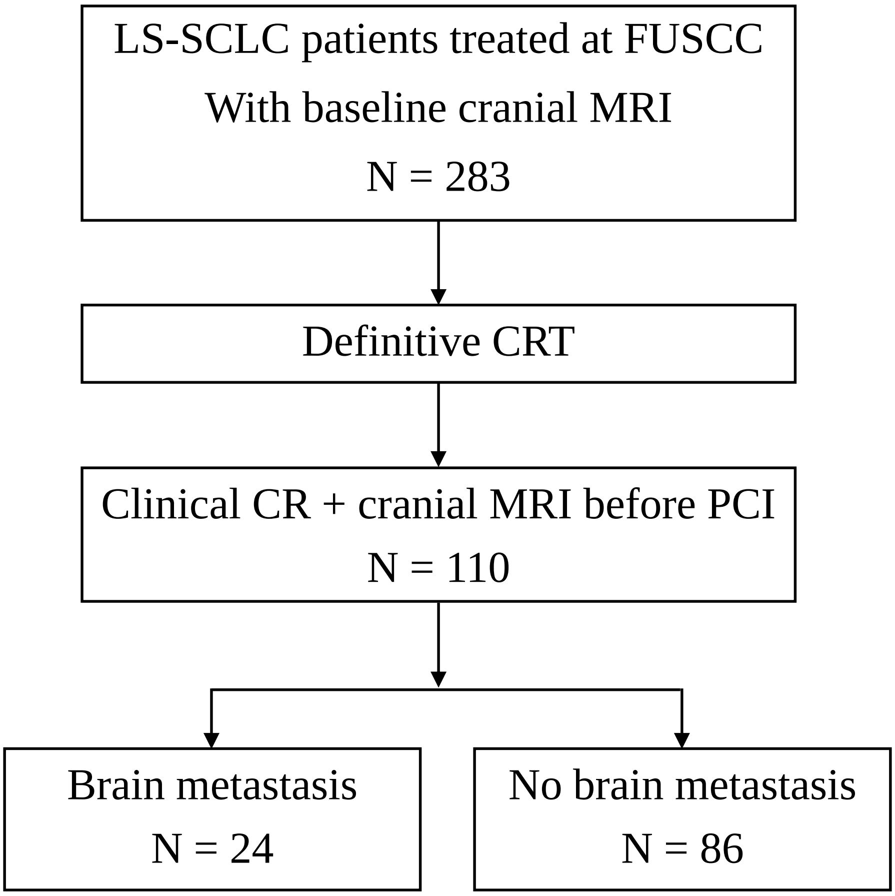


**Supplemental Figure 1. Patient selection workflow.** Selection of LS-SCLC patients treated at Fudan University Shanghai Cancer Center (FUSCC) according to the response to definitive chemoradiotherapy (CRT) and the findings of the contrast-enhanced cranial MRI performed immediately before PCI.
